# Psychosocial Assessment of Self-Harm Patients and Risk of Repeat Presentation: An Instrumental Variable Analysis Using Time of Hospital Presentation

**DOI:** 10.1371/journal.pone.0149713

**Published:** 2016-02-26

**Authors:** Robert Carroll, Chris Metcalfe, Sarah Steeg, Neil M. Davies, Jayne Cooper, Nav Kapur, David Gunnell

**Affiliations:** 1 School of Social and Community Medicine, University of Bristol, Bristol, United Kingdom; 2 Centre for Suicide Prevention, University of Manchester, Manchester, United Kingdom; 3 Manchester Mental Health and Social Care Trust, Manchester, United Kingdom; 4 Medical Research Council Integrative Epidemiology Unit, University of Bristol, Bristol, United Kingdom; 5 The National Institute for Health Research Collaboration for Leadership in Applied Health Research and Care West (NIHR CLAHRC West) at University Hospitals Bristol NHS Foundation Trust, Bristol, United Kingdom; San Raffaele Scientific Institute, ITALY

## Abstract

**Background:**

Clinical guidelines have recommended psychosocial assessment of self-harm patients for years, yet estimates of its impact on the risk of repeat self-harm vary. Assessing the association of psychosocial assessment with risk of repeat self-harm is challenging due to the effects of confounding by indication.

**Methods:**

We analysed data from a cohort study of 15,113 patients presenting to the emergency departments of three UK hospitals to investigate the association of psychosocial assessment with risk of repeat hospital presentation for self-harm. Time of day of hospital presentation was used as an instrument for psychosocial assessment, attempting to control for confounding by indication.

**Results:**

Conventional regression analysis suggested psychosocial assessment was not associated with risk of repeat self-harm within 12 months (Risk Difference (RD) 0.00 95% confidence interval (95%CI) -0.01 to 0.02). In contrast, IV analysis suggested risk of repeat self-harm was reduced by 18% (RD -0.18, 95%CI -0.32 to -0.03) in those patients receiving a psychosocial assessment. However, the instrument of time of day did not remove all potential effects of confounding by indication, suggesting the IV effect estimate may be biased.

**Conclusions:**

We found that psychosocial assessments reduce risk of repeat self-harm. This is in-line with other non-randomised studies based on populations in which allocation to assessment was less subject to confounding by indication. However, as our instrument did not fully balance important confounders across time of day, the IV effect estimate should be interpreted with caution.

## Introduction

Recent estimates suggest the risk of suicide in people who present to hospital following self-harm is 50 times that of the general population.[1] Providing effective, evidence based clinical care for this high-risk patient population is therefore a key means of reducing their risk of subsequent self-harm and suicide.

Mental health professionals use psychosocial assessments to evaluate the risk and needs of people who present to hospital following self-harm. The assessment commonly includes a discussion of the precipitating factors leading up to the episode, identification of demographic risk factors and any psychiatric co-morbidities, and the formulation of a plan for subsequent care.[2] Expert opinion suggests that assessment of such patients by a mental health specialist is a key element of their effective clinical care. Clinical guidelines in the UK reflect this with recommendations suggesting that all patients should receive a psychosocial assessment.[2, 3] Hence, psychosocial assessment has become an established part of clinical care.

Despite the prominence given to psychosocial assessment in clinical guidelines, evidence varies regarding its association with risk of repeat self-harm. Whilst it is a common sense approach to the management of self-harm patients and estimates from some observational studies have suggested a protective effect of assessment on repeat self-harm, findings from others studies do not exclude a harmful effect.[4] Effect estimates from observational studies that are consistent with harm may be a reflection of the “high risk” approach to assessment used in some centres, where only patients considered to be at greatest risk of adverse outcomes such as suicide, receive an assessment.[5] The bias introduced through this problem of confounding by indication in these settings means conventional approaches to evaluating the effect of psychosocial assessment on risk of repeat self-harm are limited. Evidence of the benefit of psychosocial assessment, and the magnitude of that benefit, is important in guiding commissioners in whether or not to invest in services that ensure comprehensive provision of mental health liaison services for hospital presenting self-harm patients.

Instrumental variables are an alternative to multi-variable adjusted regression for overcoming confounding by indication. Instruments utilise naturally occurring random variation in an exposure and mimic the conditions of an RCT. One of the major challenges of this approach is identifying a valid instrument. An instrument must be strongly related to the exposure (e.g. the likelihood of psychosocial assessment), not related to confounders (e.g. risk profile of patients), and have no direct effect on the outcome (e.g. repeat self-harm) other than through its effect on the exposure.[6] Examples of effective instruments used in health care research include geographic location, where a patient’s proximity to services is used to investigate variation in treatment;[7, 8] physicians’ preferences for prescribing one drug or another is used to predict treatment;[9] and naturally occurring variation in treatments over time.[10, 11]

We investigated whether time of day of hospital presentation is a valid instrument for identifying the effects of psychosocial assessment on risk of repeat self-harm. Provision of hospital services can vary over time and from day to day, and previous studies have used this to identify the effects of treatments.[11] In many hospitals, the availability of specialist mental health staff to undertake psychosocial assessments of self-harm patients is limited, especially out of normal working hours.[12] In the current study, we used naturally occurring variation in a patient’s likelihood of being assessed depending on the time of day they present to the Emergency Department throughout the 24-hour day (high during office hours / low out-of hours) to estimate the effects of psychosocial assessment on the risk of repeat self-harm.

## Methods

### Study population

The Manchester Self-harm (MaSH) project prospectively collected information since 1997 on all patients who presented with self-harm to three hospitals providing acute care in Manchester[13], regardless of whether or not they were admitted to a hospital inpatient bed. The MaSH project was reviewed by the South Manchester Research Ethics Committee and was deemed not to require ethical approval as the monitoring is conducted as part of a clinical audit system. All centres with the MaSH project have approval under section 251 of the NHS Act (2006) to collect patient identifiable data without patient consent. Patient records were de-identified prior to analysis. For the purposes of data collection, self-harm is defined as intentional self-poisoning or self-injury, irrespective of motivation.[14] We restricted the analysis to patients who were 16 years old or over and attended hospital between 2003 and 2010. Patients were followed-up for repeat self-harm presentations until the end of 2011. We focused on data from 2003 onwards because before 2003 information was only collected on patients who received a psychosocial assessment.

### Data collection

Specialist mental health and emergency department staff recorded the MaSH data. This information included demographic details such as age, sex, employment status, method of self-harm and the time of presentation. Where available, the staff also recorded information on the clinical characteristics of the episode of self-harm such as the patients’ previous and current medical and psychiatric history, including previous contact with services. This more detailed information was more commonly available for patients who received an assessment than those who did not receive a psychosocial assessment.

### Instrument

We investigated variation in the likelihood of psychosocial assessment across the 24-hour day as a potential instrument. Previous research had suggested self-harm patients presenting outside of Monday-Friday 9am-5pm are less likely to receive a psychosocial assessment.[12] This is likely to be due to limited provision of specialist mental health staff out-of-hours. We coded time of day as a binary variable with two periods (05:00 to 12:59 hrs/13:00 to 04:59 hrs) where receipt of psychosocial assessment was more/less likely. We defined these periods by examining the proportion of patients receiving a psychosocial assessment during each hour of the day.

### Exposure and outcome

The exposure was psychosocial assessment. A psychosocial assessment involves a mental health professional assessing both the needs and risk of the patient and making appropriate referrals for aftercare in the community. Details of psychosocial assessment are outlined in the UK’s National Institute for Clinical Excellence guidelines (CCG16) on the short term management of self-harm.[2] A mental health nurse or a psychiatrist within the liaison psychiatry service assesses most patients. The remaining patients are assessed either by another professional (e.g. an on call member of the local community mental health team) or have a joint assessment involving two of these healthcare professionals.

Our primary outcome was repeat hospital presentation for self-harm. We identified repeat episodes by checking the database for patient demographic details to see whether they had a subsequent attendance in any of the three hospitals in the 12-months after an index presentation.

### Analysis

We used two statistical approaches to estimate the effect of psychosocial assessment on risk of repeat self-harm 1) ordinary least squares regression and 2) instrumental variable regression. We recorded whether the patient had another episode of hospital presenting self-harm within one year using a binary variable. Ordinary least squares (OLS) linear regression estimates risk differences (RD) for binary outcomes.[15] We used robust standard errors to account for the non-normality of this outcome.[16] We used OLS rather than logistic regression because OLS estimates have the same scale as the instrumental variable estimates making comparison between the two approaches simpler.

Altogether, we used 4 different OLS regression models: 1) a univariable model based on the entire cohort; 2) a multivariable model controlling for well reported (<1% missing data) confounders (age, sex, method of self-harm, study centre), which minimised loss of observations due to missing data; 3) a univariable model excluding all participants with missing data; 4) a multivariable model controlling for all measured confounders (age, sex, method of self-harm, study centre, employment status, use of benzodiazepine, use of alcohol, medical risk, previous self-harm, previous psychiatric treatment, current psychiatric treatment). We choose confounding factors based on their well-established association both with risk of repeat self-harm and the likelihood of assessment.[12, 17]

We estimated the risk of repeat self-harm using instrumental variable analysis. Because our outcome was binary we used additive structural mean models based upon the assumption that patients who presented between 13:00 and 4:59 and were assessed, would also have been assessed had they presented between 5:00 and 12:59.[18, 19] We used the Stata command “ivreg2”.[19, 20] [19,20] (Baum et al., 2003; Clarke and Windmeijer, 2012) (Baum et al., 2003; Clarke and Windmeijer, 2012) (Baum *et al*., 2003, Clarke and Windmeijer, 2012) (Baum *et al*., 2003) This performs a 2-stage least squares regression analysis. The first stage estimates the association of the instrument (time of day) and the exposure (psychosocial assessment). The second stage estimates the association of the outcome (repeat self-harm) and the predicted likelihood of assessment from the first stage. If time of day is not related to the confounding factors and has no direct effect on the outcome but is associated with exposure then the instrumental variable estimate of the causal effects of psychosocial assessment will be unbiased.

### Testing instrumental variable assumptions

We tested the strength of the association of the instrument and exposure using an F-statistic. The success of the instrument in balancing measured confounders was assessed using prevalence difference ratios (PDR).[21] The PDR compares the percentage difference in the prevalence of confounders between levels of the instrument and exposure. The PDR is calculated by:

$$PDR=\;\frac{Ε\left[\text{U}|\text{Z}=1\right]-\text{E}\left[\text{U}|\text{Z}=0\right]}{Ε\left[\text{U}\left|\text{X}=1]-\text{E}[\text{U}\right|\text{X}=0\right]}$$

where U = is a potential confounder, Z = instrument (time of day), X = exposure (receipt of psychosocial assessment). The prevalence difference ratio indicates how well the instrument balances the measured confounders. If the prevalence difference ratio is large it indicates that the prevalence of the confounder is less balanced between levels of the instrument than the actual treatment (e.g there is a bigger difference by time of day (the instrument) for previous self-harm (confounder) than for psychosocial assessment (treatment)). If the prevalence difference ratio is greater than the strength of the instrument, then the IV estimate may be more biased than the adjusted OLS estimate.[9, 21]

We used Stata version 13.0 to conduct all analyses (Stata Corp, College Station TX, 2011). We calculated robust confidence intervals for the risk differences generated from the OLS and IV analysis.[16] We assessed whether the estimates from the OLS analysis differed from the 2SLS analysis using a Hausman test.[22] The null hypothesis of the Hausman test is that there are no differences between the OLS and IV estimates.

## Results

### Cohort Characteristics

Altogether, 15,633 individuals aged 16 or over presented for self-harm to one of three hospital trusts contributing data to the Manchester Self-harm Project between 2003 and 2010. Of these, 3% (476/15,633) did not have a time of presentation recorded and a further 0.3% (44/15,157) were excluded from the analysis as they did not have information on age, sex, method of self-harm or hospital. This left a total of 15,113 patients for inclusion in the main analysis. A total of 7,710 patients had no missing data in any field, these patients were included in the full multivariable analysis and the demographic details of these patients are described in Table 1.

**Table 1:** Demographic characteristics of self-harm presentations recorded at three acute trusts in Manchester.

	Full dataset	Complete cases only
	n = 15113	n = 7710
Male	6607 (43.7)	3145 (40.8)
Age > = 35	5871 (38.8)	2936 (38.1)
Psychosocial assessment	6252 (41.4)	4827 (62.6)
Method of self-harm		
*Self-poisoning*	12313 (81.5)	6609 (85.7)
*Self-injury*	2042 (13.5)	855 (11.1)
*Other*	758 (5.0)	246 (3.2)
Unemployed	-	5084 (65.9)
Benzodiazepine use	-	632 (8.2)
Used Alcohol	-	4183 (54.3)
Medical risk		
*Low*	-	3589 (46.5)
*Moderate*	-	3251 (42.2)
*High*	-	870 (11.3)
Previous SH	-	3901 (50.6)
Previous psychiatric treatment	-	3259 (42.3)
Current psychiatric treatment	-	2945 (38.2)

Overall, males were in the minority in the cohort (Full cohort: 43.7% male) and about a third were 35 or over (Full cohort 38.8% > = 35years, Complete cases cohort 38.1% > = 35years, Table 1). Self-poisoning was the most common presentation.

Patients who were assessed were more likely to have complete data on potential confounders. Altogether, two thirds (62.6%, 4827/7710) of patients with complete data received a psychosocial assessment during their presentation; the assessment rate was lower in the full cohort (41.4%). The proportion of patients with a repeat episode of hospital presenting self-harm within 12 months of their index presentation was 14.9% in both the full and complete cases cohorts.

### Conventional and instrumental variable regression

**Fig 1:**
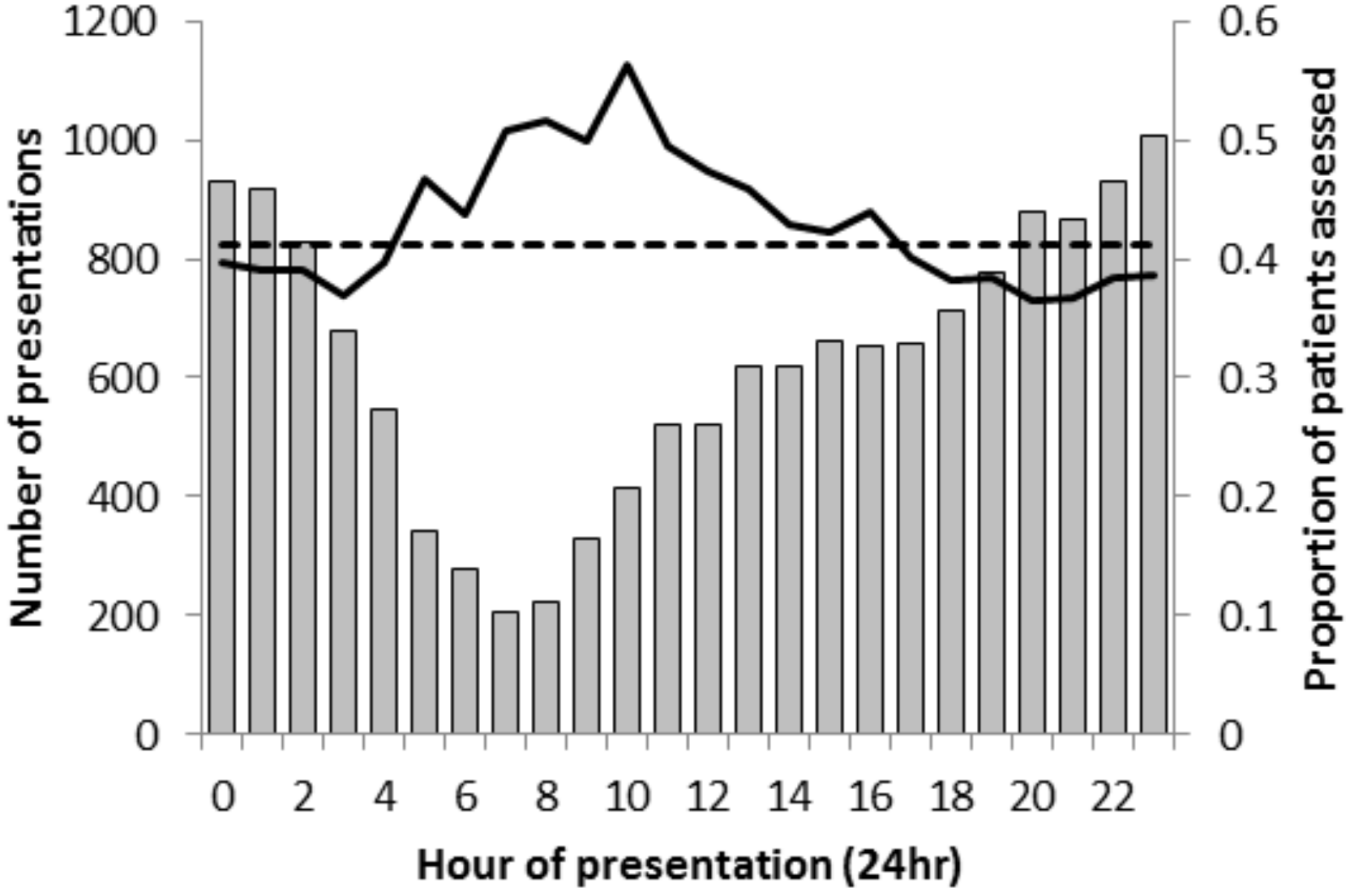
Number of people presenting for self-harm (n = 15,113) per hour over the course of a day (grey bars) by the proportion being psychosocially assessed (solid black line; and the dotted black line indicates the overall mean proportion of patients assessed).

Time of day was strongly associated with the likelihood of assessment. Rates of assessment were higher amongst patients presenting in the early to mid-morning (Fig 1). We define the period between 5:00 and 12:59 as the time when the patients were more likely to receive an assessment. The instrument was therefore binary, with a value of 1 given to patients presenting during this period. Overall, the proportion of patients being assessed was 10.0% higher between 5:00 and 12:59 (49.5% [1402/2832] assessed) than between 13:00–4:59 (39.5% [4850/12,281] assessed).

We found little evidence to suggest that patients who received an assessment had a lower risk of repeat self-harm within one year using the standard OLS regression analysis (Table 2). This was the case both in the analysis based on the full cohort (n = 15,113) and the analysis based on those patients with complete information on possible confounders (n = 7710).

**Table 2:** Psychosocial assessment of hospital presenting self-harm patients and risk of repeat self-harm within 12-months using ordinary least squares linear regression and instrumental variable two-stage least squares regression.

	n	Risk Difference^3^ (95% CI)	P	F-test^4^	Hausman test (P-value)^5^
**Ordinary least squares**					
Univariable—all available data.	15113	0.00 (-0.01 to 0.02)	0.550	-	-
Multivariable^1^—all available data	15113	0.00 (-0.01 to 0.02)	0.421	-	-
Univariable—complete cases	7710	0.01 (-0.01 to 0.02)	0.477	-	-
Multivariable^2^—complete cases	7710	0.00 (-0.02 to 0.02)	0.856	-	-
**Instrumental variable**					
Univariable—all available data.	15113	-0.18 (-0.32 to -0.03)	0.017	91.1	0.011
Multivariable^1^—all available data	15113	-0.17 (-0.31 to -0.03)	0.017	98.0	0.012
Univariable—complete cases	7710	-0.19 (-0.41 to 0.03)	0.085	47.6	0.065
Multivariable^2^—complete cases	7710	-0.14 (-0.35 to 0.08)	0.207	48.5	0.191

^1.)^ This multivariable model only includes confounding variables with minimal missing data (age, sex and method of self-harm).

^2.)^ This multivariable model adjusts for all confounding variables described in Table 1.

^3.)^ A positive risk difference indicates psychosocial assessment is associated with increased risk of repeat self-harm, a negative RD indicates a decrease in risk of repeat self-harm.

^4.)^ F-test gives an indication of the strength of the association between the instrument and the exposure.

^5.)^ The null hypothesis of the Hausman test is that the ordinary least squares RD and the IV RD are the same in the population.

In the IV analysis based on the full dataset (n = 15,113), psychosocial assessment did appear to decrease the risk of repeat self-harm (RD -0.18, 95%CI -0.32 to -0.03). This effect was similar in the instrumental variable model adjusted for confounding effects of age, sex and method (Table 2 row 6) (RD -0.17 95%CI -0.31 to -0.03). The F-test suggested time of day was strongly associated with the exposure (F = 91.1, univariable—all available data) and we found evidence of differences between the OLS and IV effect estimates using the Hausman test. In the restricted dataset with complete information on all confounders (n = 7,710), the fully adjusted model indicated an attenuated effect estimate (RD -0.14 95%CI -0.35 to 0.08). The attenuation of the effect estimate in the fully adjusted IV analysis was predominately related to controlling for the effects of previous self-harm (this reduced the effect estimate from -0.19 [95%CI -0.41 to 0.03] to -0.15 [95%CI -0.36 to 0.05]).

### Effectiveness of the instrument

Patients who were assessed appeared to differ in a number of characteristics compared to those who were not assessed (Table 3). In particular, patients who received psychosocial assessments had more often used self-poisoning as a method of self-harm, been classified as high medical risk and had a history of previous self-harm. The instrument of time of day appeared to address some of these differences, for instance, the prevalence of self-injury was similar across the periods (11.1% vs. 11.0%), as was the prevalence of current psychiatric treatment (38.16% vs. 38.36%).

**Table 3:** Confounding variables cross-tabulated by psychosocial assessment (exposure) and time of day (instrument).

	Assessment		Time of day		PDR^a^
	% No (X = 0)	% Yes (X = 1)	% 13:00–4:59 (Z = 0)	% 5:00–12:59 (Z = 1)	
Age > = 35	34.76	40.07	37.90	38.81	17%
Female	59.97	58.75	59.54	57.90	134%
Unemployed	36.14	32.82	34.79	31.14	110%
Benzodiazepine use	8.12	8.25	8.48	7.09	-1069%
Self-poisoning	81.17	88.44	85.92	84.91	-14%
Self-injury	14.95	8.78	11.11	11.03	1%
Other	3.88	2.78	2.97	4.06	-99%
Used Alcohol	50.02	56.78	55.40	49.71	-84%
*Medical risk*					
Low	56.64	40.52	46.91	45.13	11%
Moderate	35.31	46.26	42.33	41.52	-7%
High	8.05	13.22	10.76	13.35	50%
Previous SH	47.49	52.45	51.14	48.42	-55%
Previous psychiatric tx	43.43	41.58	41.79	44.17	-129%
Current psychiatric tx	35.41	39.86	38.16	38.36	4%

^a)^ PDR—Prevalence difference ratio calculated as [U|Z = 1]-[U|Z = 0] / [U|X = 1]-[U|X = 0], where U = risk factor, Z = instrument, X = assessed. The PDR should ideally be less than the strength of the instrument (9.2% in the current analysis).

The PDR associated with current psychiatric treatment (4%; Table 3) was less than the strength of the IV (9.2%), illustrating the ability of the instrument to balance this important confounder. However the prevalence of both unemployment (31.1% vs. 34.8%, χ^2^ = 7.3, P = 0.007; Table 3) and previous self-harm (48.4% vs. 51.1%, χ^2^ = 3.7, P = 0.055) differed during the two periods of the day. The PDR for unemployment was 110% and for previous self-harm was -55% (Table 3). The frequency of alcohol consumption also varied at different times of day (Table 3). Residual confounding by indication is likely to bias the unadjusted IV estimates because these PDRs were greater than the strength of the instrument. However, adjusting for this imbalance only moderately attenuated the estimated effects.

### Sensitivity analysis: IV analysis by hospital

**Fig 2:**
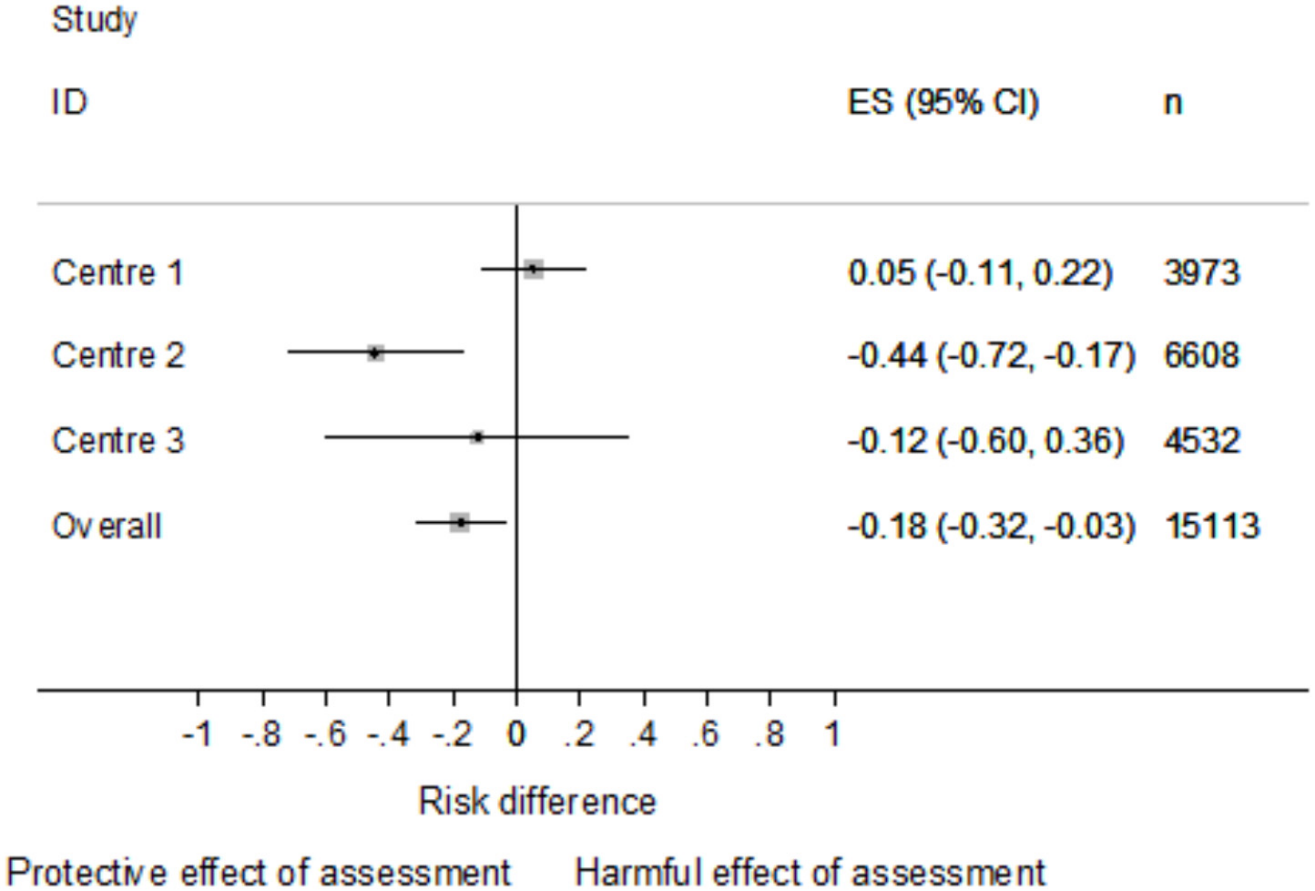
Instrumental variable estimates* of the risk difference in repeat self-harm between those who did, and did not receive a psychosocial assessment.* The overall estimate in this figure is the overall instrumental variable estimate, not the pooled estimate from a meta-analysis of the three individual centre estimates.

Temporal variation in the likelihood of receiving an assessment differed across the three centres contributing data to the MaSH project (S1 Fig). Variation in rates of assessment at different times of day was most pronounced within centre 1 and least pronounced in centre 3. The F-statistics associated with the IV analysis undertaken by centre further illustrated this: centre 1 F-test = 69.6; centre 2 F test = 31.7; centre 3 F-test = 9.3). In centre 1, where the instrument was strongest, the estimated effect of psychosocial assessment was consistent with chance (RD 0.05 95%CI -0.11 to 0.21, Fig 2) and was no different to the OLS estimate (Hausman p = 0.481). In centre 2 the instrument performed moderately well and the effect estimate based on data from this centre was in the same direction as that of the overall analysis, suggesting psychosocial assessment reduced the risk of repeat self-harm (RD -0.44 (95%CI -0.72 to -0.17, Fig 2). The instrument was less robustly associated to rates of psychosocial assessment in centre 3 and the instrumental variable based estimate (RD -0.12 95%CI -0.60 to 0.36, Fig 2) was consistent with the null.

In view of this variation across centres we undertook a random effect meta-analysis of the different effect estimates. The overall (RD -0.18, 95% CI -0.32 to -0.03) and centre specific IV estimates are summarised in Fig 2. There was statistical evidence of heterogeneity between the three centre specific estimates (χ^2^ = 9.4, df = 2, p = 0.009).

As with the overall analysis (see Table 3), when looking at each centre in turn the prevalence of some confounders were unbalanced by the instrument of time of day (S1 Table). Focusing on centre 2, the hospital with the strongest evidence of an effect of psychosocial assessment, past self-harm was balanced with a PDR of 11%–similar to the level of variation induced in the exposure by time of day (7%). However, the key confounders that had been unbalanced in the overall analysis, such as alcohol and employment, continued to be associated with high prevalence difference ratios. This was also true in the other centres, most notably with regard to the prevalence of previous self-harm, alcohol use and unemployment.

## Discussion

In the current analysis, OLS regression suggested there was little effect of psychosocial assessment on risk of repeat self-harm but this estimate is likely to be biased by confounding by indication. In contrast, using an instrumental variable approach, psychosocial assessment was associated with an 18% reduction in the risk of subsequent repeat self-harm. While the direction of the overall effect seen in the current IV analysis suggested psychosocial assessment reduces the risk of future self-harm, this effect estimate may be unreliable, as important confounders remained unbalanced across different levels of the instrument of time of day. The strength of the instrument of time of day differed across the three hospitals that contributed data to the analyses. Subsequent stratification of the analysis by centre only moderately improved the balance of confounders across time of day and therefore did not lead to a more robust estimate of the effect of psychosocial assessment on risk of repeat self-harm.

### Current findings

IV analysis, unlike the OLS analysis, suggested patients who received a psychosocial assessment had a lower risk of repeat self-harm in the year after an index presentation. The F-statistic associated with this analysis suggested time of day was strongly associated with the exposure (psychosocial assessment). However, the prevalence difference ratios for confounders such as unemployment and alcohol use indicated that the instrument did not fully balance all the measured confounders across different times of day. This inability of the instrument to balance confounders violates one of the key assumptions of IV analysis; that the IV is not associated to potential confounding factors. Controlling for these unbalanced confounders only moderately attenuated the observed protective effect of psychosocial assessment, providing some reassurance of the effect estimate’s validity although residual confounding by unmeasured factors may persist.[23]

We stratified the analysis by centre because the data came from three different hospital trusts, each operating their own psychiatric liaison service. The strength of the instrument varied across the centres. This is likely a reflection of variation in service provision (e.g. the number of staff / hours worked by liaison psychiatry services). The estimated effect of psychosocial assessment on risk of repeat self-harm also varied across the different centres contributing data. In Centre 1 the instrument was strong, but the IV analysis suggested psychosocial assessment was not associated with risk of repeat self-harm. In Centre 2 the instrument was moderately strong, but the IV analysis suggested a strong protective effect of assessment. In Centre 3 the instrument was very weakly associated with the exposure and therefore added little information.

The difference in findings between centre 1 and 2 is unexpected and could be related to a number of factors. One possible explanation may be that the confounding structures across the two centres may differ. However, the prevalence of different confounders did not differ across the two centres dramatically (see appendix B). An alternative explanation may be that the effects of psychosocial assessments genuinely differs across the two centres. It is well recognised that there are large variations in the management of self-harm and the provision of self-harm services differs between hospitals.[24] These differences include variation in the frequency of medical admission, psychosocial assessment and arrangements for aftercare (i.e. community mental health follow-up or GP referral).[24] This variation may also include variation in the delivery of health care interventions following assessment due to differences in the professional expertise of the assessor and the referral options available to them. There is some evidence for such differences between centres in Manchester (Personal communication from Dr Cooper, Aug 2014). Patients attending centre 2 are offered follow-up in the community and additional psychological therapy. These interventions are not available at the other centres and may explain the present heterogeneity in effect estimates between hospitals.

### Strength and limitations

This analysis is strengthened by the large sample size and rich prospective data collected as part of the MaSH project.[25] The MaSH project collects data on self-harm patients from multiple sources, including hospital records, liaison psychiatry records, as well as information from the local mental health trust. These data sources allow the collection of a large number of important confounders of the association of psychosocial assessment and risk of repeat self-harm, including current and previous psychiatric service use, detailed information regarding methods of self-harm, and the treatment received during a patient’s hospital presentation. Information of this detail is not routinely available in administrative health care datasets.

The MaSH project also collects detailed data from three different hospitals within Manchester. An additional strength to these data is that the three hospitals represent a well-defined catchment area, therefore minimising the chance of under-reporting of repeat clinical presentations.

The main weakness of this analysis was the presence of residual confounding. Important confounders were still unbalanced at different times of day, suggesting that other unmeasured confounders that were not included in the multivariable model may still confound the observed protective association between psychosocial assessment and risk of repeat self-harm.

### Relevance to other studies

While psychosocial assessment of self-harm patients has been recommend since 2004 by both NICE and the Royal College of Psychiatrists,[2, 3] robust observational evidence demonstrating its effectiveness in reducing the incidence of repeat self-harm has remained elusive. Estimating its effect may be particularly challenging in centres where only a small proportion of high-risk patients receive the intervention. This high-risk approach to assessment leads to considerable confounding by indication and an apparent increase in the risk of repeat self-harm in people who are assessed. This is because, in centres assessing relatively few patients, only patients who are likely to repeat self-harm (i.e. high risk patients), such as those with a previous history of psychiatric treatment, are targeted for assessment.[4]

In a previous observational analysis of data from the multicentre study of self-harm in the UK, a protective effect of psychosocial assessment was observed in centres where higher proportions of patients were assessed (Oxford: HR 0.59, 95%CI 0.52 to 0.68; Derby: HR 0.59, 95%CI 0.48 to 0.74). However, in Manchester, where a high-risk approach to assessment is employed (46.7% assessed in Manchester compared to 75.7% and 67.9% in Oxford and Derby respectively), the effect of assessment on repetition was consistent with chance (Manchester: HR 0.99, CI 0.90 to 1.09). We replicated this latter finding in the current analysis when we used ordinary least squares. In contrast, our instrumental variable based estimate suggested a protective effect of assessment which is in-line with estimates from other cohorts that are based on patients presenting to services where a high-risk approach to assessment is not used. While the instrumental variable estimate is likely unreliable due to residual confounding, it is consistent with results from other centres that are not so heavily affected by confounding by indication.

Confounding by indication has been described as a most stubborn form of bias, a bias that is particularly difficult to completely control for in observational settings.[26] The reliability of the current instrumental variable based effect estimate is limited due to imbalances in confounders across different times of day and should therefore be interpreted with caution. Nonetheless, our findings provide some further evidence of psychosocial assessment’s protective effect on risk of repeat self-harm and are in-line with effect estimates from observational studies where treatment was less confounded by indication. Future research into the effectiveness of this intervention should consider including data from more centres and the use of alternative instruments, such as physicians’ preference.

## Supporting Information

S1 Fig Number of people presenting for self-harm over the course of a day per hour (grey bars) by the proportion being psychosocially assessed (solid black line; the dotted black line indicates the overall mean proportion of patients assessed). (TIF)

S1 Table Prevalence difference ratios associated with self-harm patient demographics. (DOCX)
