# Supplementary figures and images for: Psychosocial Assessment of Self-Harm Patients and Risk of Repeat Presentation: An Instrumental Variable Analysis Using Time of Hospital Presentation
Source: PLoS One. 2016 Feb 26;11(2):e0149713. doi: 10.1371/journal.pone.0149713 (PMC4769277; doi:10.1371/journal.pone.0149713)

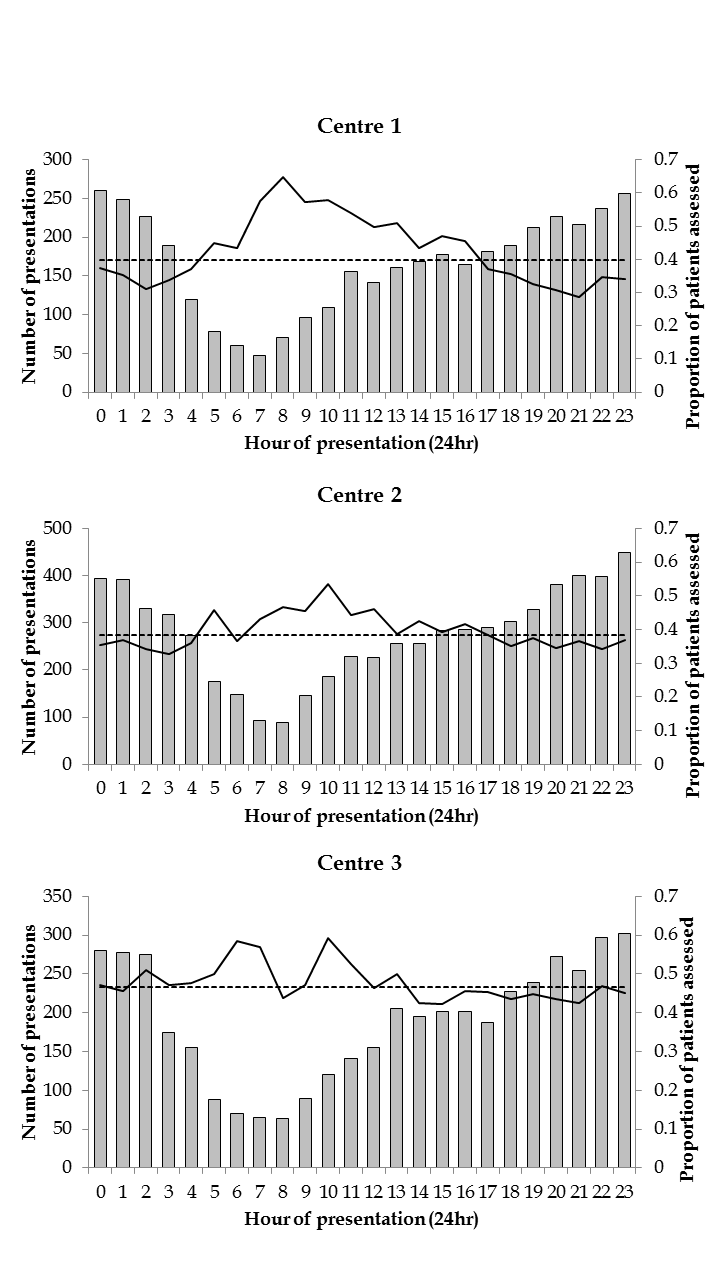

Supplement: S1 Fig — (TIF) [file pone.0149713.s001.tif]
