# Supplementary material for: Psychosocial Assessment of Self-Harm Patients and Risk of Repeat Presentation: An Instrumental Variable Analysis Using Time of Hospital Presentation
Source: PLoS One. 2016 Feb 26;11(2):e0149713. doi: 10.1371/journal.pone.0149713 (PMC4769277; doi:10.1371/journal.pone.0149713)
Supplement: S1 Table — (DOCX) [file pone.0149713.s002.docx]

| Web appendix - S1 Table  S1 Table. Prevalence difference ratios associated with self-harm patient demographics. | | | | | | | | | | | | |  |  |  |
| --- | --- | --- | --- | --- | --- | --- | --- | --- | --- | --- | --- | --- | --- | --- | --- |
|  | **Centre 1** | | | | | **Centre 2** | | | | | **Centre 3** | | | | |
|  | **Assessment** | | **Time of day** | | **PDR^1^** | **Assessment** | | **Time of day** | | **PDR^1^** | **Assessment** | | **Time of day** | | **PDR^1^** |
|  | **No** | **Yes** | **13:00-4:59 (Z=0)** | **5:00-12:59 (Z=1)** |  | **No** | **Yes** | **13:00-4:59 (Z=0)** | **5:00-12:59 (Z=1)** |  | **No** | **Yes** | **13:00-4:59 (Z=0)** | **5:00-12:59 (Z=1)** |  |
| Age >=35 | 39.6 | 45.7 | 43.9 | 42.7 | -20% | 26.9 | 34.6 | 31.1 | 33.8 | 34% | 39.5 | 41.8 | 40.6 | 42.1 | 69% |
|  |  |  |  |  |  |  |  |  |  |  |  |  |  |  |  |
| Female | 60.5 | 56.6 | 57.8 | 58.3 | -14% | 59.2 | 59.4 | 59.9 | 57.2 | -1827% | 60.4 | 59.6 | 60.2 | 58.4 | 232% |
|  |  |  |  |  |  |  |  |  |  |  |  |  |  |  |  |
| Unemployed | 43.1 | 36.4 | 39.6 | 35.0 | 69% | 36.9 | 36.1 | 37.1 | 34.2 | 372% | 31.9 | 27.0 | 29.9 | 24.9 | 101% |
|  |  |  |  |  |  |  |  |  |  |  |  |  |  |  |  |
| Benzodiazepine use | 8.3 | 9.1 | 9.2 | 7.3 | -248% | 6.6 | 8.1 | 7.8 | 6.7 | -66% | 9.5 | 7.8 | 8.7 | 7.4 | 75% |
|  |  |  |  |  |  |  |  |  |  |  |  |  |  |  |  |
| Self-poisoning | 85.4 | 91.0 | 89.1 | 89.5 | 8% | 79.4 | 84.1 | 82.2 | 82.8 | 12% | 80.6 | 91.1 | 87.4 | 84.2 | -31% |
| Self-injury | 11.8 | 6.7 | 8.4 | 8.3 | 1% | 17.0 | 12.7 | 14.7 | 13.0 | 41% | 14.7 | 6.2 | 9.4 | 10.6 | -14% |
| Other | 2.8 | 2.3 | 2.5 | 2.2 | 69% | 3.6 | 3.2 | 3.1 | 4.2 | -256% | 4.7 | 2.7 | 3.2 | 5.2 | -105% |
|  |  |  |  |  |  |  |  |  |  |  |  |  |  |  |  |
| Used Alcohol | 51.9 | 59.0 | 58.2 | 50.8 | -104% | 49.7 | 53.6 | 52.6 | 50.6 | -51% | 49.3 | 58.4 | 56.3 | 47.9 | -93% |
|  |  |  |  |  |  |  |  |  |  |  |  |  |  |  |  |
| Medical risk |  |  |  |  |  |  |  |  |  |  |  |  |  |  |  |
| Low | 60.7 | 40.9 | 47.3 | 47.9 | -3% | 58.5 | 44.0 | 50.0 | 47.3 | 18% | 52.9 | 36.8 | 43.8 | 40.6 | 20% |
| Moderate | 33.2 | 48.1 | 43.8 | 40.9 | -20% | 34.4 | 45.7 | 41.6 | 40.9 | -6% | 37.2 | 45.6 | 42.1 | 42.7 | 6% |
| High | 6.1 | 11.0 | 9.0 | 11.3 | 48% | 7.1 | 10.4 | 8.4 | 11.7 | 102% | 9.9 | 17.6 | 14.0 | 16.7 | 34% |
|  |  |  |  |  |  |  |  |  |  |  |  |  |  |  |  |
| Previous SH | 45.9 | 51.3 | 50.3 | 46.8 | -65% | 47.8 | 55.1 | 52.5 | 51.7 | -11% | 48.0 | 50.6 | 50.4 | 45.6 | -183% |
|  |  |  |  |  |  |  |  |  |  |  |  |  |  |  |  |
| Previous psychiatric tx | 45.1 | 37.5 | 40.2 | 39.0 | 16% | 40.1 | 43.3 | 41.7 | 43.8 | 65% | 45.6 | 42.6 | 42.8 | 48.2 | -184% |
|  |  |  |  |  |  |  |  |  |  |  |  |  |  |  |  |
| Current psychiatric tx | 35.0 | 37.6 | 37.0 | 35.8 | -48% | 30.3 | 39.4 | 35.8 | 36.7 | 11% | 40.3 | 41.9 | 41.0 | 42.1 | 69% |
| 1. PDR - Prevalence difference ration; calculated via [U\|Z=1]-[U\|Z=0] / [U\|X=1]-[U\|X=0], where U = risk factor, Z = instrument, X = assessed | | | | | | | | | | | | | | | |
